# Two new species of *Dacne* Latreille (Coleoptera, Erotylidae) from China, with a key to Chinese species and subspecies of *Dacne*

**DOI:** 10.3897/zookeys.261.4495

**Published:** 2013-01-24

**Authors:** Cong-Chao Dai, Mei-Jun Zhao

**Affiliations:** 1 Department of Biology, College of Life and Environmental Sciences, Shanghai Normal University, Shanghai, 200234, P. R. China

**Keywords:** Coleoptera, Erotylidae, *Dacne*, *Xenodacne*, identification key, new species, China

## Abstract

Two new species *Dacne (Xenodacne) tangliangi* **sp. n.** and*Dacne (Xenodacne) hujiayaoi* **sp. n.** are described from China. A key to Chinese species and subspecies of genus *Dacne* Latreille is provided.

## Introduction

The genus *Dacne* Latreille is considered to be one of the most primitive members of the subfamily Erotylinae (Wegrzynowicz 2002; Leschen 2003). Skelley (1997) reviewed this genus and later updated a world checklist and key (Skelley 2003). In general, little is known about *Dacne* in the Orient. Some work has been done in neighboring countries (Arrow 1925; Chûjô 1969; Chûjô and Chûjô 1988; Narukawa 1992; Chûjô and Lee 1993; Nikitsky and Kompantzev 1995), but nothing focuses specifically on China. Previously, only two species and one subspecies have been reported from China, *Dacne (Dacne) picta* Crotch (1873) (Fig. 18), *Dacne (Dacne) japonica* Crotch (1873) (Fig. 16) and *Dacne (Xenodacne) zonaria taiwana* Chûjô (1976) (picture of this subspecies is not available for the present study)

In thus work, two new species of the genus *Dacne* are described and illustrated: *Dacne (Xenodacne) tangliangi* sp. n. and *Dacne (Xenodacne) enodacne*) *hujiayaoi* sp. n.from Yunnan Province, China.

## Material and methods

The specimens examined in this paper were collected in a wide variety of woodland fungi, in crevices under bark or in other retreats by splitting and sifting. For an examination of the male genitalia, the abdominal segments were detached from the body after softening in hot water. The aedeagi, together with other dissected parts, were mounted in Euparal (Chroma Gesellschaft Schmidt, Koengen, Germany) on plastic slides. Photos of sexual characters were taken with a Canon G9 camera attached to an Olympus SZX 16 stereoscope; habitus photos were taken with a Canon macro photo lens MP-E 65 mm attached to a Canon EOS7D camera.

The specimens treated in this study are deposited in the following public collections:

**SHNU** Department of Biology, Shanghai Normal University, P. R. China

**FSCA **Florida State Collection of Arthropods, USA [Paul E. Skelley]

## Taxonomy

### Key to Chinese species and subspecies of *Dacne*

Parts of the following key were taken from Skelley (2003).

**Table:** 

1	Pronotal lateral margin thin for entire length; pronotum swollen anteriorly, projecting forward beyond anterior pronotal angles	2
–	Pronotal lateral margin thickened, often broader anteriorly; pronotal anterior margin normal, not projecting forward beyond anterior angles	3
2	Pronotum with darkened disc	*Dacne (Dacne) picta* Crotch
–	Pronotum entirely orange	*Dacne (Dacne) japonica* Crotch
3	Each elytron with one orange mark	*Dacne (Xenodacne) tanglian*giDai & Zhao, sp. n.
–	Each elytron with two orange markings	4
4	Body shining; Legs black with tarsi dark brown	*Dacne (Xenodacne) zonaria taiwana* Chûjô
–	Body indistinctly shining; legs reddish-brown	*Dacne (Xenodacne) hujiayaoi* Dai & Zhao, sp. n.

#### Dacne (Xenodacne) tangliangi

Dai & Zhao sp. n.

urn:lsid:zoobank.org:act:EF23E61E-5D0C-4859-8059-8E4851B4B2CD

http://species-id.net/wiki/Dacne_tangliangi

Figs. 1, 2 3–9 19

##### Type material.

**Holotype:** **CHINA: Yunnan Prov.:** ♂, Nabanhe N.R., Bengganghan, Nanmugahe, 22°06'N, 100°27'E, alt. 1700 m, 13.XI.2008, H Jia-Yao & TANG Liang leg. (SHNU). **Paratypes:** **CHINA: Yunnan Prov.:** 4♂♂, 4♀♀, same data as holotype (SHNU); 1♂, 1♀, same data as holotype (FSCA).

##### Description.

**Figures 1–2.**
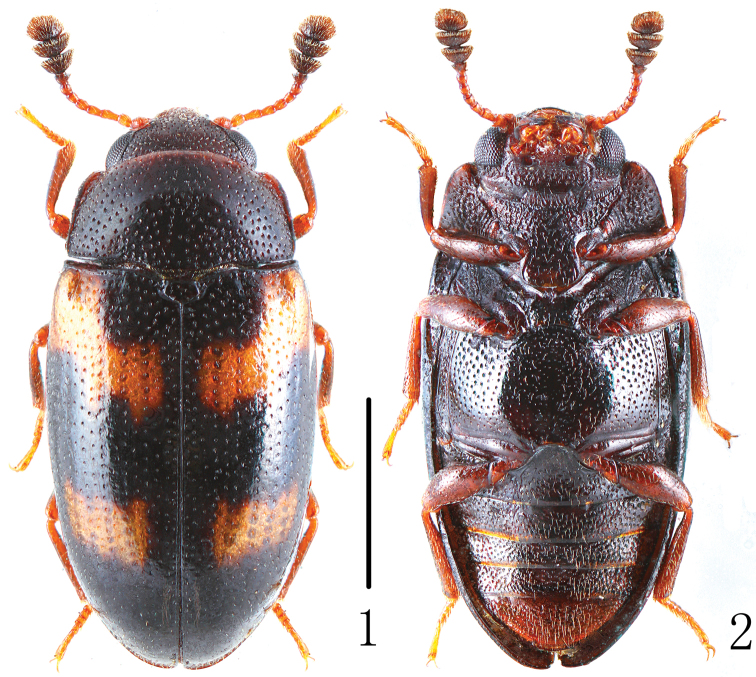
Habitus of *Dacne (Xenodacne) tangliangi* in dorsal and ventral view. Scale = 1 mm.

Body (Fig. 1, 2) stout, elongate, length: 2.8–3.1 mm; width: 1.29–1.40 mm. Head and elytra black; pronotum general black with reddish-brown sides; legs, palpi and base of antennae reddish-brown; antennal club dark brown. Each elytron with two orange bands.

Head width between eyes = 4 times eye diameter in dorsal view; punctation coarse, sparse, separated by 3-4 puncture diameters; epistome truncate, lacking marginal line on anterior margin; stridulatory files not evident. Antennae (Fig. 9) long, extending behind posterior border of pronotum; antennomere III about 1.4 times as long as IV; antennomere VIII slightly wider than VII, about 1.2 times as wide as long; antennomere IX trapezoidal; antennomere X transverse; antennomere XI almost elliptic; relative lengths of antennomeres II–XI: 12.5: 13.5: 8.5: 8.0: 8.0: 8.0: 8.0: 11.0: 11.0: 14.0. Maxillary and labial terminal palpomeres acuminate, sensory area restricted to apex. Mentum broad with anterior projection, almost triangular, slightly more than 2 times wider than long.

Pronotum arched, widest at base (pl/pw = 0.61–0.65); slightly narrowing toward apex; lateral margin thickened anteriorly; pronotal anterior margin normal, not projecting forward beyond anterior angles (typical for the subgenus *Xenodacne*). Pronotum distinctly punctured medially, finely and closely punctured laterally.

Prosternum with anterior edge straight, lacking marginal bead; posterior process broad, width more than diameter of procoxa; prosternal lines apparently lacking; punctures coarse and close, diameter = eye facet, separated by 1-2 puncture diameters. Abdomen with distinct coxal lines on first ventrite nearly attaining posterior margin. Legs with tibia not dilated at apex.

Scutellum pentagonal, finely and sparely punctured.

Elytra margined basally; widest at middle, then gradually narrowing to apex; with fine punctures.

**Figures 3–9.**
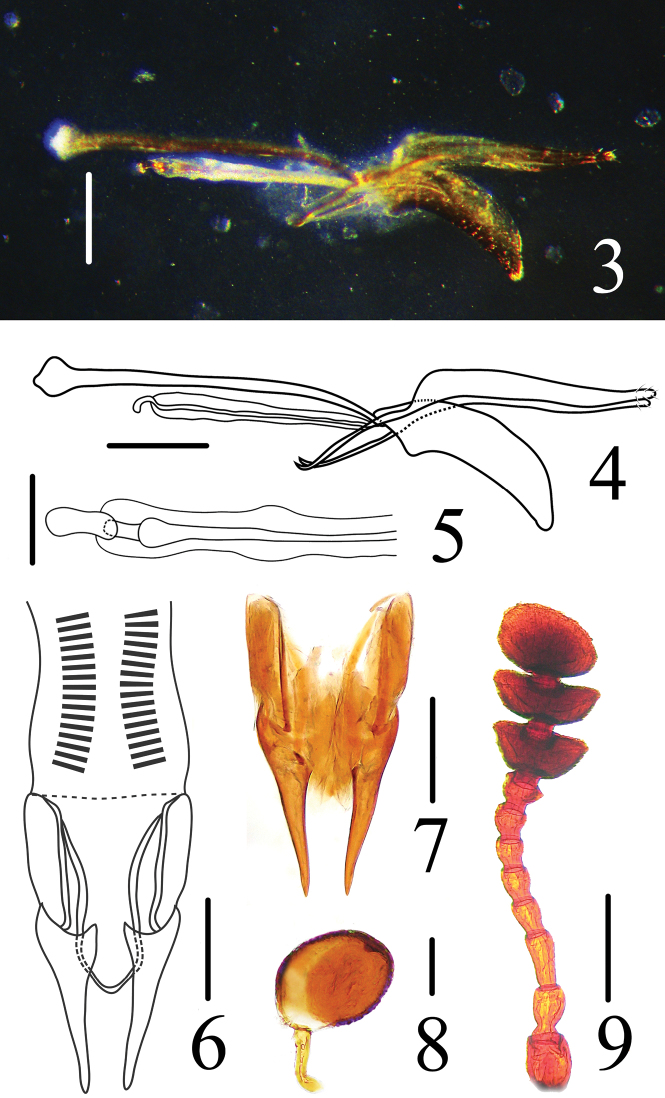
*Dacne (Xenodacne) tangliangi*. **3, 4** aedeagus in lateral views **5** internal sac and flagellum in dorsal view **6, 7** female genitalia in ventral views **8** female spermatheca **9** antenna. Scales = 0.05 mm(**5, 8**), Scales = 0.2 mm(**3, 4, 6, 7, 9**).

Male genitalia (Fig. 3, 4) moderately curved; median lobe short, apically pointed; median strut long, about 1.8 times as long as median lobe. Tegmen with parameres long, flattened, tightly fitting basal piece and each other. Internal sac simple (Fig. 5).

Female genitalia (Fig. 6, 7) with reduced stylus; coxite apically and curved terminally, chisel-like, length nearly equal to valvifer; paraproct narrowed apically; female spermatheca (Fig. 8) with head almost round shaped.

##### Distribution.

China (Yunnan Province).

**Diagnosis.** *Dacne tangliangi* is most similar to *Dacne (Xenodacne) maculata*Chûjô due to similar form and color pattern of the body. *Dacne tangliangi* can be distinguished from *Dacne maculata* by the black pronotum, scutellum not transverse (length/width<1.5), posterior band in elytron not extending to the border and occurs in southwest China. *Dacne maculata* has a reddish pronotum, scutellum transverse (length/width>1.5), posterior band in elytron extending to the border and occurs in Japan and Siberia (Chûjô and Chûjô 1988).

##### Etymology.

This species is named in honor of Mr. Liang Tang, collector of the new species and teacher of the senior author.

#### Dacne (Xenodacne) hujiayaoi

Dai & Zhao sp. n.

urn:lsid:zoobank.org:act:E9574B20-FA53-4C5F-AB2C-B44A7BDC35AA

http://species-id.net/wiki/Dacne_hujiayaoi

**Figures 10–11.**
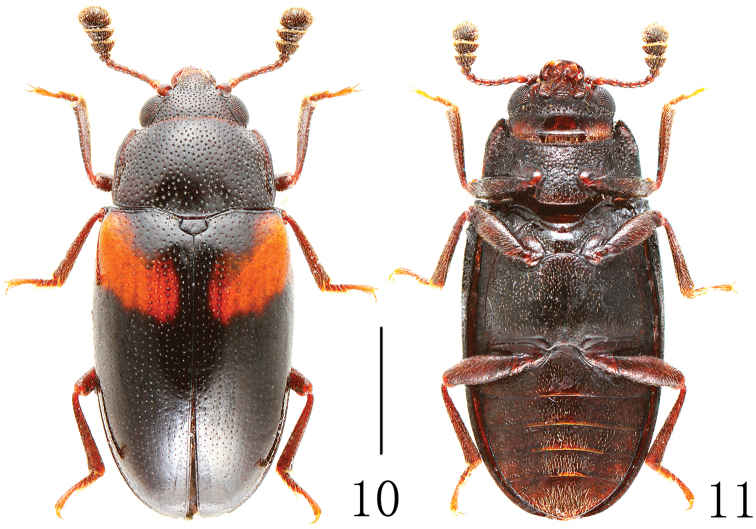
Habitus of *Dacne (Xenodacne) hujiayaoi* in dorsal and ventral view. Scale = 1 mm.

Figs. 10, 11 12–15 17

##### Type material.

**Holotype:** **CHINA: Yunnan Prov.:** ♂, Nabanhe N.R., Bengganghan, Nanmugahe, 22°06'N, 100°27'E, alt. 1700 m, 13.XI.2008, H Jia-Yao & TANG Liang leg. (SHNU).

##### Description.

Body (Fig. 10, 11) stout, elongate, length: 3.6 mm; width: 1.5 mm. Body black; legs, palpi and base of antennae reddish-brown; antennal club dark brown. Each elytron with one orange band.

Head width between eyes = 3.5 times eye diameter in dorsal view; punctation coarse, separated by 1-3 puncture diameters; epistome truncate, lacking marginal line on anterior margin; stridulatory files not evident. Antennae (Fig. 15) long, extending behind posterior border of pronotum; antennomere III about 1.2 times as long as IV; antennomere VIII slightly wider than VII, about 1.5 times as wide as long; antennomere IX trapezoidal; antennomere X transverse; antennomere XI almost elliptic; relative lengths of antennomeres II–XI: 9.0: 11.5: 8.0: 8.0: 8.0: 8.0: 7.5: 10.0: 10.0: 17.0. Maxillary and labial terminal palpomeres acuminate, sensory area restricted to apex. Mentum broad with anterior projection, almost triangular, slightly more than 1.5 times wider than long.

Pronotum arched, widest at base (pl/pw = 0.62); slightly narrowing toward apex; lateral margin thickened anteriorly; pronotal anterior margin normal, not projecting forward beyond anterior angles (typical for the subgenus *Xenodacne*). Pronotum distinctly punctured medially, finely and closely punctured laterally.

Prosternum with anterior edge straight, lacking marginal bead; posterior process broad, width more than diameter of procoxa; prosternal lines apparently lacking; punctures coarse and close, diameter = eye facet, separated by 0.5-1.0 puncture diameters. Abdomen with distinct coxal lines on first ventrite nearly attaining posterior margin. Legs with tibia not dilated at apex.

Scutellum pentagonal, finely and sparely punctured.

Elytra margined basally; widest at middle, then gradually narrowing to apex; with fine punctures.

**Figures 12–15.**
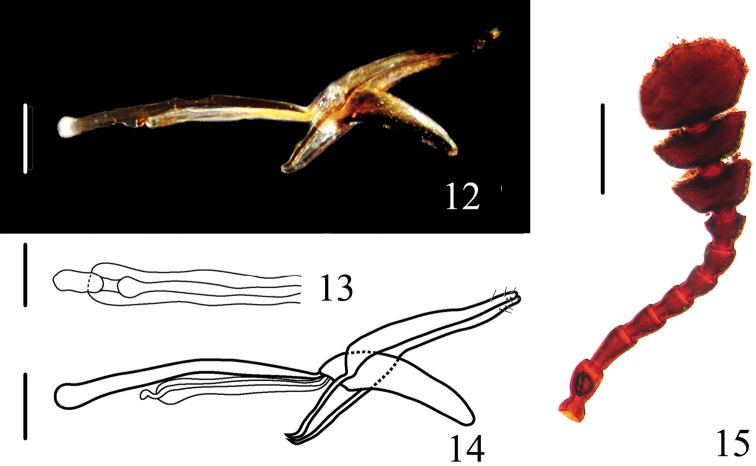
*Dacne (Xenodacne) hujiayaoi*. **12, 14** aedeagus in lateral views **13** internal sac and flagellum in dorsal view **15** antenna. Scales = 0.05 mm(**13**), Scales = 0.2 mm(**12, 14, 15**).

Male genitalia (Fig. 12, 14) moderately curved; median lobe short, apically pointed; median strut long, about 1.6 times as long as median lobe. Tegmen with parameres long, flattened, tightly fitting basal piece and each other. Internal sac simple (Fig. 13).

##### Distribution.

China (Yunnan Province).

##### Diagnosis.

*Dacne hujiayaoi* is most similar to *Dacne (Xenodacne) zonaria* Lewis and it’s subspecies due to similar form and color pattern of the body. *Dacne hujiayaoi* can be distinguished from *Dacne zonaria* by body indistinctly shining, eyes large (head width between eyes = 3.5 times eye diameter in dorsal view), the reddish-brown legs and occurs in southwest China. *Dacne zonaria* has the body distinctly shining, eyes small (head width between eyes > 4 times eye diameter in dorsal view), the black legs and occurs in Japan, Korea, Siberia and Taiwan (Chûjô and Chûjô 1988).

##### Etymology.

This species is named in honor of Mr. Jia-Yao Hu, collector of the new species and teacher of the senior author.

**Figures 16–19.**
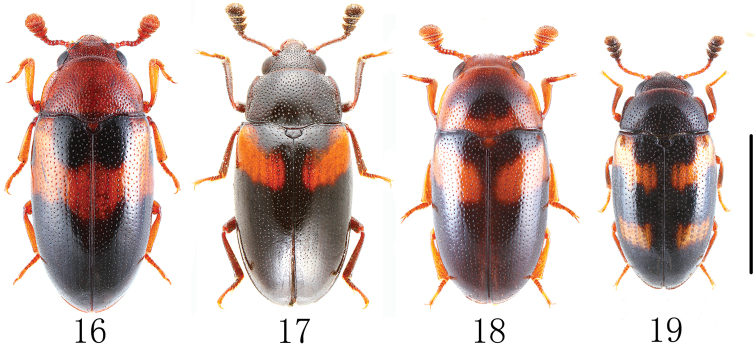
Habitus of Chinese species of *Dacne* in dorsal view (*Dacne zonaria taiwana* is excluded). **16** *Dacne (Dacne) japonica* **17** *Dacne (Xenodacne) hujiayaoi* **18** *Dacne (Dacne) picta* **19** *Dacne (Xenodacne) tangliangi*. Scale = 2 mm.

## Supplementary Material

XML Treatment for Dacne (Xenodacne) tangliangi

XML Treatment for Dacne (Xenodacne) hujiayaoi
